# Supplementary material for: Predicting the Pathway Involvement of Metabolites in Both Pathway Categories and Individual Pathways
Source: bioRxiv. 2024 Aug 9:2024.08.07.607025. Preprint. [Version 1] doi: 10.1101/2024.08.07.607025 (PMC11326255; doi:10.1101/2024.08.07.607025)
Supplement: Supplement 1 [file NIHPP2024.08.07.607025v1-supplement-1.pdf]

*Table S1 – Model performance metrics for each dataset.*

| Subset | Scaled | Metric | Mean Score | Standard deviation |
|---|---|---|---|---|
| Combined | True | Accuracy | 0.9930 | 0.0011 |
| | | F1 score | 0.8013 | 0.0223 |
| | | MCC | 0.7997 | 0.0209 |
| | | Precision | 0.7551 | 0.0396 |
| | | Recall | 0.8551 | 0.0129 |
| | False | Accuracy | 0.9926 | 0.0003 |
| | | F1 score | 0.7743 | 0.0085 |
| | | MCC | 0.7706 | 0.0086 |
| | | Precision | 0.7735 | 0.0176 |
| | | Recall | 0.7754 | 0.0130 |
| Level 2 | True | Accuracy | 0.9473 | 0.0082 |
| | | F1 score | 0.7556 | 0.0262 |
| | | MCC | 0.7275 | 0.0294 |
| | | Precision | 0.7528 | 0.0575 |
| | | Recall | 0.7629 | 0.0340 |
| | * False | Accuracy | 0.9592 | 0.0027 |
| | | F1 score | 0.8069 | 0.0114 |
| | | MCC | 0.7844 | 0.0129 |
| | | Precision | 0.8128 | 0.0224 |
| | | Recall | 0.8019 | 0.0193 |
| Level 3 | True | Accuracy | 0.9930 | 0.0015 |
| | | F1 score | 0.6529 | 0.0359 |
| | | MCC | 0.6547 | 0.0314 |
| | | Precision | 0.6830 | 0.0883 |
| | | Recall | 0.6440 | 0.0821 |
| | False | Accuracy | 0.9902 | 0.0041 |
| | | F1 score | 0.6101 | 0.0628 |
| | | MCC | 0.6182 | 0.0481 |
| | | Precision | 0.5593 | 0.1238 |
| | | Recall | 0.7129 | 0.0787 |
| ** The results highlighted in yellow were provided by the work of Huckvale and Moseley in the supplemental materials of the manuscript of DOI: https://doi.org/10.3390/metabo14050266* | | | | |

*Table S2 - Sum of True/False Positives/Negative metrics across CV iterations by pathway hierarchy level (L2 or L3). Results are from training on the combined and scaled dataset.*

| Subset | Pathway Hierarchy Level | Scaled | Metric | Sum | Count (number of pathway categories times 100 CV iterations) |
|---|---|---|---|---|---|

| | | | | | |
|---|---|---|---|---|---|
| Combined | 2 | TRUE | True positives | 69,342 | 1,200 |
| | | | True negatives | 597,569 | 1,200 |
| | | | False positives | 12,142 | 1,200 |
| | | | False negatives | 2,963 | 1,200 |
| | | FALSE | True positives | 63,596 | 1,200 |
| | | | True negatives | 599,093 | 1,200 |
| | | | False positives | 10,963 | 1,200 |
| | | | False negatives | 8,677 | 1,200 |
| | 3 | TRUE | True positives | 77,484 | 17,200 |
| | | | True negatives | 9,639,265 | 17,200 |
| | | | False positives | 36,124 | 17,200 |
| | | | False negatives | 21,911 | 17,200 |
| | | FALSE | True positives | 69,546 | 17,200 |
| | | | True negatives | 9,646,907 | 17,200 |
| | | | False positives | 28,137 | 17,200 |
| | | | False negatives | 29,881 | 17,200 |

*Table S3 – Characteristics and performance metrics for each L2 and L3 pathway. Results derive from training on the combined and scaled dataset.*

| Pathway ID | Number of metabolites | Number of non-hydrogen atoms | MCC score | True positives | True negatives | False positives | False negatives |
|---|---|---|---|---|---|---|---|
| Amino acid metabolism | 611 | 10774 | 0.8409598893071340 | 5792.0 | 49225.0 | 1555.0 | 391.0 |
| Biosynthesis of other secondary metabolites | 1486 | 38836 | 0.9081988788786460 | 14491.0 | 40591.0 | 1735.0 | 382.0 |
| Carbohydrate metabolism | 514 | 11485 | 0.8902161978132910 | 4837.0 | 50831.0 | 898.0 | 195.0 |
| Chemical structure transformation maps | 437 | 9298 | 0.7442351678288250 | 3885.0 | 50243.0 | 1999.0 | 483.0 |
| Energy metabolism | 173 | 4470 | 0.8441864642903980 | 1539.0 | 54626.0 | 398.0 | 157.0 |
| Glycan biosynthesis and metabolism | 324 | 18936 | 0.8687650948335800 | 3133.0 | 52902.0 | 730.0 | 172.0 |
| Lipid metabolism | 677 | 22159 | 0.9480287144475790 | 6788.0 | 49618.0 | 535.0 | 119.0 |
| Metabolism of cofactors and vitamins | 549 | 15569 | 0.8192698358391200 | 5099.0 | 49542.0 | 1800.0 | 269.0 |
| Metabolism of other amino acids | 274 | 4989 | 0.8341470754511110 | 2554.0 | 53293.0 | 760.0 | 217.0 |
| Metabolism of terpenoids and polyketides | 1093 | 34676 | 0.9402708096069460 | 10590.0 | 44929.0 | 756.0 | 317.0 |
| Nucleotide metabolism | 169 | 3592 | 0.9420564036585730 | 1650.0 | 55260.0 | 166.0 | 33.0 |
| Xenobiotics biodegradation and metabolism | 939 | 16780 | 0.9349955392946330 | 8984.0 | 46509.0 | 810.0 | 228.0 |
| 00010 Glycolysis / Gluconeogenesis | 43 | 997 | 0.7521514556232700 | 349.0 | 55995.0 | 129.0 | 99.0 |
| 00020 Citrate cycle (TCA cycle) | 42 | 1089 | 0.7736618071841690 | 346.0 | 56150.0 | 142.0 | 62.0 |
| 00030 Pentose phosphate pathway | 65 | 1189 | 0.5801059320016230 | 424.0 | 55899.0 | 425.0 | 194.0 |

| | | | | | | | |
|---|---|---|---|---|---|---|---|
| **00040  Pentose and glucuronate interconversions** | 69 | 1256 | 0.5896851476187490 | 457.0 | 55574.0 | 410.0 | 223.0 |
| **00051  Fructose and mannose metabolism** | 71 | 1506 | 0.5425171605375680 | 424.0 | 55891.0 | 370.0 | 328.0 |
| **00052  Galactose metabolism** | 64 | 1330 | 0.578369297572232 | 410.0 | 55245.0 | 381.0 | 214.0 |
| **00053  Ascorbate and aldarate metabolism** | 71 | 1325 | 0.5480372306228640 | 465.0 | 56062.0 | 517.0 | 251.0 |
| **00061  Fatty acid biosynthesis** | 23 | 670 | 0.5857307522988000 | 202.0 | 56524.0 | 270.0 | 48.0 |
| **00062  Fatty acid elongation** | 39 | 2179 | 0.6824945492484340 | 347.0 | 55567.0 | 339.0 | 27.0 |
| **00071  Fatty acid degradation** | 51 | 2484 | 0.6439211962872030 | 437.0 | 56211.0 | 423.0 | 92.0 |
| **00073  Cutin, suberine and wax biosynthesis** | 23 | 656 | 0.6142145231530840 | 185.0 | 56583.0 | 223.0 | 36.0 |
| **00100  Steroid biosynthesis** | 68 | 2069 | 0.8438381240718750 | 616.0 | 55703.0 | 131.0 | 94.0 |
| **00120  Primary bile acid biosynthesis** | 55 | 2299 | 0.8489302256003490 | 491.0 | 56404.0 | 124.0 | 51.0 |
| **00121  Secondary bile acid biosynthesis** | 36 | 1184 | 0.8496184733002620 | 345.0 | 56321.0 | 80.0 | 42.0 |
| **00130  Ubiquinone and other terpenoid-quinone biosynthesis** | 91 | 2339 | 0.7202567520407570 | 711.0 | 55463.0 | 313.0 | 228.0 |
| **00140  Steroid hormone biosynthesis** | 108 | 2745 | 0.8985746926606160 | 984.0 | 55486.0 | 163.0 | 57.0 |
| **00190  Oxidative phosphorylation** | 14 | 372 | 0.5561799510943540 | 81.0 | 56711.0 | 88.0 | 44.0 |
| **00195  Photosynthesis** | 6 | 184 | 0.5687909308210510 | 43.0 | 56722.0 | 49.0 | 19.0 |
| **00220  Arginine biosynthesis** | 29 | 605 | 0.7568809031535640 | 214.0 | 56472.0 | 49.0 | 89.0 |
| **00230  Purine metabolism** | 121 | 2755 | 0.8532967940978590 | 1212.0 | 55166.0 | 366.0 | 56.0 |
| **00232  Caffeine metabolism** | 28 | 563 | 0.8541798746855700 | 242.0 | 56951.0 | 26.0 | 57.0 |
| **00240  Pyrimidine metabolism** | 90 | 1787 | 0.8113729465430260 | 837.0 | 55378.0 | 293.0 | 97.0 |
| **00250  Alanine, aspartate and glutamate metabolism** | 51 | 1075 | 0.6216987663625870 | 310.0 | 56068.0 | 188.0 | 184.0 |
| **00253  Tetracycline biosynthesis** | 46 | 1468 | 0.7919846416467210 | 412.0 | 56190.0 | 192.0 | 34.0 |
| **00254  Aflatoxin biosynthesis** | 36 | 1093 | 0.9055626098952720 | 337.0 | 56389.0 | 44.0 | 26.0 |
| **00260  Glycine, serine and threonine metabolism** | 60 | 1074 | 0.632184821848565 | 397.0 | 56010.0 | 229.0 | 225.0 |
| **00261  Monobactam biosynthesis** | 49 | 1098 | 0.7165231174376000 | 353.0 | 56369.0 | 142.0 | 134.0 |
| **00270  Cysteine and methionine metabolism** | 96 | 1699 | 0.6882694148003660 | 721.0 | 55366.0 | 376.0 | 263.0 |
| **00280  Valine, leucine and isoleucine degradation** | 52 | 1633 | 0.6458800086230890 | 393.0 | 56501.0 | 311.0 | 127.0 |
| **00290  Valine, leucine and isoleucine biosynthesis** | 29 | 503 | 0.7687005955828100 | 241.0 | 56030.0 | 70.0 | 74.0 |
| **00300  Lysine biosynthesis** | 55 | 1231 | 0.675042799256142 | 391.0 | 55833.0 | 222.0 | 151.0 |
| **00310  Lysine degradation** | 70 | 1565 | 0.6013072452787590 | 428.0 | 55910.0 | 284.0 | 272.0 |
| **00311  Penicillin and cephalosporin biosynthesis** | 31 | 692 | 0.7169593911073640 | 222.0 | 56107.0 | 83.0 | 91.0 |
| **00330  Arginine and proline metabolism** | 77 | 1316 | 0.624971876536468 | 555.0 | 55507.0 | 468.0 | 202.0 |
| **00331  Clavulanic acid biosynthesis** | 17 | 319 | 0.5018821415057870 | 90.0 | 56570.0 | 69.0 | 111.0 |
| **00332  Carbapenem biosynthesis** | 45 | 1047 | 0.7562328936259140 | 348.0 | 56338.0 | 107.0 | 115.0 |

| | | | | | | | |
|---|---|---|---|---|---|---|---|
| **00333** Prodigiosin biosynthesis | 29 | 784 | 0.7242515796302220 | 227.0 | 56171.0 | 98.0 | 74.0 |
| **00340** Histidine metabolism | 61 | 1115 | 0.7109610395634370 | 449.0 | 55834.0 | 212.0 | 149.0 |
| **00350** Tyrosine metabolism | 95 | 1600 | 0.7132958932262360 | 720.0 | 55563.0 | 365.0 | 206.0 |
| **00360** Phenylalanine metabolism | 61 | 1463 | 0.5784794345723250 | 353.0 | 55826.0 | 288.0 | 219.0 |
| **00361** Chlorocyclohexane and chlorobenzene degradation | 91 | 1206 | 0.784074944506343 | 769.0 | 55253.0 | 249.0 | 167.0 |
| **00362** Benzoate degradation | 97 | 2614 | 0.6105447143364770 | 648.0 | 55191.0 | 490.0 | 319.0 |
| **00363** Bisphenol degradation | 29 | 568 | 0.869213773716127 | 240.0 | 56730.0 | 43.0 | 29.0 |
| **00364** Fluorobenzoate degradation | 40 | 817 | 0.7277840822453640 | 271.0 | 56572.0 | 81.0 | 121.0 |
| **00365** Furfural degradation | 16 | 428 | 0.6696526147960390 | 113.0 | 56955.0 | 55.0 | 56.0 |
| **00380** Tryptophan metabolism | 109 | 2126 | 0.7957896057720780 | 941.0 | 55135.0 | 262.0 | 209.0 |
| **00400** Phenylalanine, tyrosine and tryptophan biosynthesis | 56 | 1015 | 0.6432877086056760 | 373.0 | 56476.0 | 183.0 | 225.0 |
| **00401** Novobiocin biosynthesis | 37 | 1012 | 0.7470854287503790 | 298.0 | 56205.0 | 107.0 | 93.0 |
| **00402** Benzoxazinoid biosynthesis | 16 | 291 | 0.7176017065480430 | 102.0 | 56445.0 | 40.0 | 40.0 |
| **00403** Indole diterpene alkaloid biosynthesis | 38 | 1301 | 0.8302009318289570 | 325.0 | 56047.0 | 96.0 | 38.0 |
| **00404** Staurosporine biosynthesis | 64 | 1779 | 0.8191649844663780 | 539.0 | 56090.0 | 133.0 | 102.0 |
| **00405** Phenazine biosynthesis | 41 | 952 | 0.6381257912091950 | 280.0 | 56366.0 | 167.0 | 147.0 |
| **00410** beta-Alanine metabolism | 45 | 973 | 0.7087123261934690 | 340.0 | 56524.0 | 158.0 | 119.0 |
| **00430** Taurine and hypotaurine metabolism | 30 | 756 | 0.7607651217055730 | 237.0 | 56470.0 | 72.0 | 76.0 |
| **00440** Phosphonate and phosphinate metabolism | 68 | 1453 | 0.7966198293069850 | 572.0 | 56491.0 | 187.0 | 103.0 |
| **00450** Selenocompound metabolism | 31 | 655 | 0.6830059208336810 | 235.0 | 56240.0 | 151.0 | 70.0 |
| **00460** Cyanoamino acid metabolism | 50 | 763 | 0.6116918933903410 | 325.0 | 56103.0 | 233.0 | 175.0 |
| **00470** D-Amino acid metabolism | 79 | 1252 | 0.6598403086147240 | 563.0 | 55805.0 | 320.0 | 249.0 |
| **00480** Glutathione metabolism | 53 | 1258 | 0.5799322025862940 | 308.0 | 56044.0 | 260.0 | 182.0 |
| **00500** Starch and sucrose metabolism | 54 | 1346 | 0.6392912010311820 | 361.0 | 56104.0 | 249.0 | 156.0 |
| **00510** N-Glycan biosynthesis | 26 | 1129 | 0.7291468418447760 | 200.0 | 56482.0 | 94.0 | 55.0 |
| **00512** Mucin type O-glycan biosynthesis | 3 | 127 | -0.00027401531865905300 | 0.0 | 56871.0 | 9.0 | 27.0 |
| **00513** Various types of N-glycan biosynthesis | 4 | 73 | 0.027205888688917800 | 1.0 | 56796.0 | 37.0 | 33.0 |
| **00514** Other types of O-glycan biosynthesis | 6 | 175 | 0.4525634034550170 | 24.0 | 56442.0 | 22.0 | 37.0 |
| **00515** Mannose type O-glycan biosynthesis | 6 | 158 | 0.39863663629348100 | 25.0 | 56606.0 | 45.0 | 31.0 |
| **00520** Amino sugar and nucleotide sugar metabolism | 137 | 4033 | 0.7401919779086580 | 1122.0 | 55133.0 | 547.0 | 230.0 |
| **00521** Streptomycin biosynthesis | 39 | 969 | 0.7195865469126920 | 294.0 | 56349.0 | 121.0 | 106.0 |
| **00522** Biosynthesis of 12-, 14- and 16-membered macrolides | 95 | 4281 | 0.8777338297470100 | 832.0 | 56092.0 | 137.0 | 91.0 |

| | | | | | | | |
|---|---|---|---|---|---|---|---|
| **00523** Polyketide sugar unit biosynthesis | 73 | 2432 | 0.8319233194762350 | 672.0 | 55971.0 | 223.0 | 53.0 |
| **00524** Neomycin, kanamycin and gentamicin biosynthesis | 103 | 3240 | 0.8811058059278500 | 892.0 | 55873.0 | 128.0 | 108.0 |
| **00525** Acarbose and validamycin biosynthesis | 52 | 1224 | 0.8125023355285470 | 446.0 | 55946.0 | 132.0 | 73.0 |
| **00531** Glycosaminoglycan degradation | 13 | 375 | 0.1117813630720040 | 13.0 | 56619.0 | 78.0 | 131.0 |
| **00532** Glycosaminoglycan biosynthesis - chondroitin sulfate / dermatan sulfate | 4 | 114 | 0.6764558122534930 | 25.0 | 56932.0 | 10.0 | 14.0 |
| **00534** Glycosaminoglycan biosynthesis - heparan sulfate / heparin | 2 | 58 | 0.49601170570954100 | 8.0 | 56530.0 | 18.0 | 2.0 |
| **00540** Lipopolysaccharide biosynthesis | 71 | 5332 | 0.7119255155083730 | 579.0 | 55988.0 | 300.0 | 165.0 |
| **00541** O-Antigen nucleotide sugar biosynthesis | 110 | 3764 | 0.8140831474534970 | 994.0 | 55373.0 | 376.0 | 84.0 |
| **00550** Peptidoglycan biosynthesis | 41 | 2517 | 0.7096009771486460 | 337.0 | 56391.0 | 199.0 | 81.0 |
| **00552** Teichoic acid biosynthesis | 40 | 2113 | 0.6129455314284880 | 292.0 | 56336.0 | 271.0 | 107.0 |
| **00561** Glycerolipid metabolism | 50 | 1585 | 0.7288252640151510 | 411.0 | 55887.0 | 202.0 | 104.0 |
| **00562** Inositol phosphate metabolism | 59 | 1456 | 0.7590277931409680 | 434.0 | 56088.0 | 130.0 | 142.0 |
| **00563** Glycosylphosphatidylinositol (GPI)-anchor biosynthesis | 10 | 298 | 0.2623363639353410 | 36.0 | 56544.0 | 148.0 | 65.0 |
| **00564** Glycerophospholipid metabolism | 76 | 1973 | 0.8329444010426630 | 686.0 | 55779.0 | 191.0 | 83.0 |
| **00565** Ether lipid metabolism | 45 | 1002 | 0.6583909820237230 | 317.0 | 56322.0 | 245.0 | 92.0 |
| **00571** Lipoarabinomannan (LAM) biosynthesis | 10 | 466 | 0.4353021170675020 | 55.0 | 56497.0 | 88.0 | 56.0 |
| **00572** Arabinogalactan biosynthesis - Mycobacterium | 9 | 474 | 0.2429860220833520 | 28.0 | 56063.0 | 113.0 | 65.0 |
| **00590** Arachidonic acid metabolism | 85 | 2186 | 0.9020556642167380 | 803.0 | 55749.0 | 144.0 | 31.0 |
| **00591** Linoleic acid metabolism | 30 | 744 | 0.6694539415743330 | 251.0 | 56492.0 | 171.0 | 80.0 |
| **00592** alpha-Linolenic acid metabolism | 62 | 2027 | 0.8213088985460710 | 529.0 | 55909.0 | 150.0 | 79.0 |
| **00600** Sphingolipid metabolism | 56 | 1650 | 0.7081600596698010 | 490.0 | 55922.0 | 329.0 | 89.0 |
| **00601** Glycosphingolipid biosynthesis - lacto and neolacto series | 26 | 2339 | 0.9355472092944740 | 255.0 | 56329.0 | 21.0 | 14.0 |
| **00603** Glycosphingolipid biosynthesis - globo and isoglobo series | 11 | 673 | 0.8199876238303620 | 93.0 | 56348.0 | 26.0 | 15.0 |
| **00604** Glycosphingolipid biosynthesis - ganglio series | 16 | 1368 | 0.8008036639157520 | 123.0 | 56686.0 | 29.0 | 32.0 |
| **00620** Pyruvate metabolism | 51 | 1324 | 0.6833139881448820 | 377.0 | 55811.0 | 189.0 | 156.0 |
| **00621** Dioxin degradation | 69 | 1294 | 0.8371759600896710 | 584.0 | 56071.0 | 102.0 | 122.0 |
| **00622** Xylene degradation | 46 | 740 | 0.7119955738202290 | 335.0 | 56375.0 | 127.0 | 141.0 |
| **00623** Toluene degradation | 56 | 1059 | 0.4611271845303230 | 272.0 | 55978.0 | 276.0 | 348.0 |
| **00624** Polycyclic aromatic hydrocarbon degradation | 121 | 2254 | 0.7537280966208090 | 950.0 | 55356.0 | 340.0 | 264.0 |
| **00625** Chloroalkane and chloroalkene degradation | 11 | 345 | 0.6468011531234290 | 75.0 | 56552.0 | 54.0 | 29.0 |
| **00626** Naphthalene degradation | 69 | 1438 | 0.6357825704670690 | 404.0 | 55875.0 | 217.0 | 238.0 |

| | | | | | | | |
|---|---|---|---|---|---|---|---|
| **00627 Aminobenzoate degradation** | 106 | 2056 | 0.6457810046377090 | 721.0 | 55168.0 | 357.0 | 411.0 |
| **00630 Glyoxylate and dicarboxylate metabolism** | 78 | 1961 | 0.6352256427893340 | 553.0 | 55918.0 | 390.0 | 237.0 |
| **00633 Nitrotoluene degradation** | 33 | 658 | 0.8075168569911570 | 244.0 | 56672.0 | 20.0 | 101.0 |
| **00640 Propanoate metabolism** | 41 | 1216 | 0.5275516914956930 | 253.0 | 56251.0 | 284.0 | 169.0 |
| **00642 Ethylbenzene degradation** | 20 | 488 | 0.4856692068933750 | 70.0 | 56518.0 | 53.0 | 98.0 |
| **00643 Styrene degradation** | 27 | 628 | 0.5442834314614210 | 143.0 | 56398.0 | 90.0 | 151.0 |
| **00650 Butanoate metabolism** | 43 | 1339 | 0.6337107369408950 | 278.0 | 56370.0 | 185.0 | 134.0 |
| **00660 C5-Branched dibasic acid metabolism** | 40 | 872 | 0.5609187135182550 | 237.0 | 56121.0 | 225.0 | 145.0 |
| **00670 One carbon pool by folate** | 42 | 1048 | 0.5127876754159670 | 242.0 | 56678.0 | 305.0 | 159.0 |
| **00680 Methane metabolism** | 102 | 2534 | 0.7533470795904140 | 856.0 | 55473.0 | 424.0 | 140.0 |
| **00710 Carbon fixation in photosynthetic organisms** | 30 | 571 | 0.6911517217454610 | 239.0 | 56530.0 | 141.0 | 74.0 |
| **00720 Carbon fixation pathways in prokaryotes** | 48 | 1706 | 0.7372234377314740 | 403.0 | 56001.0 | 193.0 | 95.0 |
| **00730 Thiamine metabolism** | 34 | 598 | 0.6351616397377490 | 209.0 | 56495.0 | 94.0 | 146.0 |
| **00740 Riboflavin metabolism** | 36 | 928 | 0.6430606851335270 | 239.0 | 56313.0 | 120.0 | 143.0 |
| **00750 Vitamin B6 metabolism** | 42 | 664 | 0.68878962694997 | 301.0 | 56341.0 | 132.0 | 137.0 |
| **00760 Nicotinate and nicotinamide metabolism** | 77 | 1312 | 0.7566014310904930 | 609.0 | 55501.0 | 212.0 | 173.0 |
| **00770 Pantothenate and CoA biosynthesis** | 53 | 975 | 0.8125071653405430 | 477.0 | 55856.0 | 163.0 | 59.0 |
| **00780 Biotin metabolism** | 29 | 704 | 0.6510390615787090 | 218.0 | 56140.0 | 173.0 | 67.0 |
| **00785 Lipoic acid metabolism** | 41 | 1160 | 0.6104343767640310 | 333.0 | 56235.0 | 337.0 | 106.0 |
| **00790 Folate biosynthesis** | 81 | 1941 | 0.7820022413017530 | 663.0 | 55408.0 | 244.0 | 123.0 |
| **00791 Atrazine degradation** | 22 | 267 | 0.8879918741580400 | 225.0 | 56344.0 | 40.0 | 17.0 |
| **00830 Retinol metabolism** | 33 | 1005 | 0.6565859869886410 | 275.0 | 56211.0 | 271.0 | 44.0 |
| **00860 Porphyrin metabolism** | 161 | 7551 | 0.7786835720932580 | 1439.0 | 54827.0 | 577.0 | 225.0 |
| **00900 Terpenoid backbone biosynthesis** | 58 | 1676 | 0.6983224177776770 | 470.0 | 55767.0 | 285.0 | 124.0 |
| **00901 Indole alkaloid biosynthesis** | 96 | 2470 | 0.8787511753723070 | 887.0 | 55879.0 | 154.0 | 87.0 |
| **00902 Monoterpenoid biosynthesis** | 70 | 1166 | 0.5985305822686890 | 412.0 | 55937.0 | 285.0 | 257.0 |
| **00903 Limonene degradation** | 56 | 1156 | 0.8168080310251450 | 433.0 | 55751.0 | 97.0 | 95.0 |
| **00904 Diterpenoid biosynthesis** | 135 | 3445 | 0.8595465627286350 | 1181.0 | 55543.0 | 237.0 | 140.0 |
| **00905 Brassinosteroid biosynthesis** | 33 | 1088 | 0.9358212201321070 | 307.0 | 56710.0 | 28.0 | 14.0 |
| **00906 Carotenoid biosynthesis** | 142 | 5553 | 0.8970744191367720 | 1348.0 | 55336.0 | 201.0 | 101.0 |
| **00907 Pinene, camphor and geraniol degradation** | 65 | 2056 | 0.7458763981009230 | 497.0 | 56128.0 | 200.0 | 135.0 |
| **00908 Zeatin biosynthesis** | 38 | 964 | 0.7921510901356950 | 312.0 | 56427.0 | 98.0 | 65.0 |
| **00909 Sesquiterpenoid and triterpenoid biosynthesis** | 92 | 2033 | 0.6272931720065450 | 635.0 | 55392.0 | 417.0 | 319.0 |
| **00910 Nitrogen metabolism** | 15 | 453 | 0.6766126048746800 | 96.0 | 56451.0 | 55.0 | 37.0 |

| | | | | | | | |
|---|---|---|---|---|---|---|---|
| **00920 Sulfur metabolism** | 52 | 1345 | 0.6747486742538950 | 332.0 | 55763.0 | 162.0 | 154.0 |
| **00930 Caprolactam degradation** | 35 | 827 | 0.5605678597768910 | 201.0 | 56555.0 | 186.0 | 128.0 |
| **00940 Phenylpropanoid biosynthesis** | 74 | 1812 | 0.7649315369779430 | 618.0 | 55988.0 | 238.0 | 138.0 |
| **00941 Flavonoid biosynthesis** | 92 | 2361 | 0.7736911928778960 | 761.0 | 55662.0 | 287.0 | 153.0 |
| **00942 Anthocyanin biosynthesis** | 79 | 3666 | 0.8521201133396060 | 702.0 | 55893.0 | 155.0 | 86.0 |
| **00943 Isoflavonoid biosynthesis** | 76 | 1902 | 0.8394004082119710 | 665.0 | 56336.0 | 149.0 | 102.0 |
| **00944 Flavone and flavonol biosynthesis** | 66 | 2332 | 0.8267688659404890 | 637.0 | 56071.0 | 210.0 | 60.0 |
| **00945 Stilbenoid, diarylheptanoid and gingerol biosynthesis** | 34 | 1108 | 0.75979790751362 | 263.0 | 56251.0 | 110.0 | 57.0 |
| **00946 Degradation of flavonoids** | 47 | 1048 | 0.48029316462370500 | 239.0 | 56494.0 | 286.0 | 224.0 |
| **00950 Isoquinoline alkaloid biosynthesis** | 144 | 3669 | 0.9095550637821420 | 1371.0 | 55811.0 | 148.0 | 117.0 |
| **00960 Tropane, piperidine and pyridine alkaloid biosynthesis** | 91 | 1891 | 0.793513754160815 | 730.0 | 55647.0 | 213.0 | 160.0 |
| **00965 Betalain biosynthesis** | 32 | 895 | 0.70684621101882 | 215.0 | 56478.0 | 87.0 | 90.0 |
| **00966 Glucosinolate biosynthesis** | 89 | 1622 | 0.8882234493070090 | 798.0 | 55783.0 | 149.0 | 51.0 |
| **00980 Metabolism of xenobiotics by cytochrome P450** | 115 | 2346 | 0.8206921470458940 | 897.0 | 55710.0 | 180.0 | 203.0 |
| **00981 Insect hormone biosynthesis** | 33 | 950 | 0.770038639507383 | 309.0 | 55970.0 | 144.0 | 45.0 |
| **00982 Drug metabolism - cytochrome P450** | 94 | 1809 | 0.9115830208550640 | 845.0 | 55678.0 | 84.0 | 77.0 |
| **00983 Drug metabolism - other enzymes** | 74 | 1677 | 0.832535891433835 | 590.0 | 56134.0 | 106.0 | 128.0 |
| **00984 Steroid degradation** | 29 | 811 | 0.780509722686123 | 245.0 | 56261.0 | 76.0 | 61.0 |
| **00996 Biosynthesis of various alkaloids** | 89 | 2537 | 0.6769750135188250 | 632.0 | 55466.0 | 351.0 | 241.0 |
| **00997 Biosynthesis of various other secondary metabolites** | 82 | 1720 | 0.620256681819166 | 581.0 | 55712.0 | 467.0 | 240.0 |
| **00998 Biosynthesis of various antibiotics** | 126 | 2586 | 0.7469649931826780 | 960.0 | 55172.0 | 320.0 | 311.0 |
| **00999 Biosynthesis of various plant secondary metabolites** | 174 | 5573 | 0.7524500635441920 | 1378.0 | 54640.0 | 512.0 | 360.0 |
| **01040 Biosynthesis of unsaturated fatty acids** | 80 | 4509 | 0.7931406916340190 | 765.0 | 55455.0 | 406.0 | 23.0 |
| **01051 Biosynthesis of ansamycins** | 48 | 1614 | 0.7428984261088510 | 389.0 | 56201.0 | 157.0 | 110.0 |
| **01052 Type I polyketide structures** | 25 | 1280 | 0.42387899618925300 | 87.0 | 56704.0 | 84.0 | 157.0 |
| **01053 Biosynthesis of siderophore group nonribosomal peptides** | 29 | 809 | 0.6271569045656970 | 183.0 | 56929.0 | 101.0 | 115.0 |
| **01054 Nonribosomal peptide structures** | 14 | 1062 | 0.6934693151337760 | 72.0 | 56579.0 | 6.0 | 66.0 |
| **01055 Biosynthesis of vancomycin group antibiotics** | 26 | 909 | 0.4828061733893560 | 129.0 | 56358.0 | 156.0 | 119.0 |
| **01056 Biosynthesis of type II polyketide backbone** | 30 | 1034 | 0.8639344763724760 | 268.0 | 56292.0 | 48.0 | 36.0 |
| **01057 Biosynthesis of type II polyketide products** | 160 | 5716 | 0.9271790666316450 | 1429.0 | 55208.0 | 119.0 | 99.0 |
| **01059 Biosynthesis of enediyne antibiotics** | 73 | 2401 | 0.7364613478070240 | 546.0 | 55934.0 | 200.0 | 184.0 |

| | | | | | | | |
|---|---|---|---|---|---|---|---|
| **01060** Biosynthesis of plant secondary metabolites | 134 | 2511 | 0.576574875334981 | 998.0 | 53880.0 | 1125.0 | 355.0 |
| **01061** Biosynthesis of phenylpropanoids | 105 | 2123 | 0.5836969274582290 | 779.0 | 55420.0 | 822.0 | 299.0 |
| **01062** Biosynthesis of terpenoids and steroids | 96 | 2135 | 0.5477297998792720 | 641.0 | 55026.0 | 727.0 | 328.0 |
| **01063** Biosynthesis of alkaloids derived from shikimate pathway | 142 | 2962 | 0.7421287384147680 | 1150.0 | 54903.0 | 546.0 | 240.0 |
| **01064** Biosynthesis of alkaloids derived from ornithine, lysine and nicotinic acid | 63 | 993 | 0.7425623581569070 | 499.0 | 55896.0 | 220.0 | 124.0 |
| **01065** Biosynthesis of alkaloids derived from histidine and purine | 34 | 565 | 0.7307892738408580 | 280.0 | 56389.0 | 141.0 | 67.0 |
| **01066** Biosynthesis of alkaloids derived from terpenoid and polyketide | 47 | 1161 | 0.5083709282611360 | 226.0 | 56450.0 | 201.0 | 230.0 |
| **01070** Biosynthesis of plant hormones | 66 | 1454 | 0.46495064652427200 | 396.0 | 55407.0 | 724.0 | 231.0 |
